# Impact of non-weight-dependent low-dose somatropin on bone accrual in childhood-onset GH deficient in the transition: an 18-month randomized controlled trial

**DOI:** 10.1016/j.jped.2024.10.010

**Published:** 2025-01-02

**Authors:** Valesca Mansur Kuba, Antonia B.S. Castro, Cláudio Leone, Durval Damiani

**Affiliations:** a Faculdade de Medicina da Universidade de São Paulo (FMUSP), Instituto da Criança, Unidade de Endocrinologia Pediátrica, São Paulo, São Paulo, Brazil; b Faculdade de Medicina de Campos, Rio de Janeiro, Rio de Janeiro, Brazil; c Faculdade de Medicina da Universidade de São Paulo (FMUSP), Escola de Saúde Pública (ESP), Departamento de Saúde Materno-Infantil, São Paulo, São Paulo, Brazil

**Keywords:** Transition phase, Somatropin, Bone mineral density, Peak bone mass, Adolescence, Osteoporosis

## Abstract

**Objective:**

Discontinuation of growth hormone therapy (rhGH) upon completion of linear growth may adversely affect bone mineral density and content (BMD/BMC) in adolescents with childhood-onset GH deficiency (CO-GHD) and predisposition to osteoporosis. Although the benefits of weight-dependent somatropin high doses over bone gain are established, little is known about fixed low doses. We analyzed the impact of non-weight-based low-dose somatropin on bone accrual during the transition among CO-DGH patients, treated since childhood.

**Methods:**

Lumbar spine (LS) and whole-body (WB) BMD and BMC were measured at baseline and after 18 months in 54 adolescents (age: 16.8 ± 1.6 years). They were retested and reclassified as GH sufficient (GHS, *n* = 28) and GH insufficient. The last group was later randomized to use rhGH (GH on; *n* = 15) or no treatment (GH off, *n* = 11) in this single-center open-label study. The average dose of rhGH was 0.5 ± 0.18 mg/day.

**Results:**

When comparing the groups, the GH off group had a lower percentage change in LS BMD than the GHS (0.53 % ± 5.9 vs. 4.42 % ± 4.1, respectively, *p* < 0.04). However, in the analysis of the GH on and off subgroups, the LS BMC percentage change was higher in the GH on (11.02 % ± 10.12 vs. 2.05 % ± 10.31, respectively, *p* < 0.04).

**Conclusion:**

Non-weight-based low-dose somatropin withdrawal for 18 months limits bone accrual in LS of CO-DGH subjects in transition, predisposing them to osteoporosis in adult life.

## Introduction

Osteoporosis is a systemic disease that compromises bone microarchitecture and leads to, bone fragility. It constitutes one of the main public health problems since after the age of 50 years, 30 % of the population will suffer some type of fracture with a high mortality rate.^1^

Childhood and adolescence are fundamental periods for the development of peak bone mass via the gradual increment of bone tissue and bone formation, two processes that predominate over bone resorption.^2^ The main action of growth hormone (GH) is to promote linear growth by increasing protein synthesis and osteoblastic activity,^3^ which makes treating GH-deficient adolescents quite challenging. Recombinant human GH (rhGH) was initially approved for treating childhood-onset GH-deficient children (CO-DGH) to help these children reach a greater height. When the final height was reached, it was then customary to discontinue the treatment.^4^ This transition phase corresponds to the period that extends from puberty to the age of 30 years when peak bone mass is reached.^5^ Several trials have shown that GH actions are far more complex than merely stimulating linear growth, with effects promoting lean mass accrual and bone mineralization. Robust evidence indicates that peak bone mass acquired in childhood and adolescence is the major determinant of fracture risk later in adulthood.^6^ This finding has led to questions about discontinuing rhGH therapy and the necessity of reassessment of the persistence of GH deficiency in transition, which can manifest as osteoporosis in GH-deficient adults (AO-DGH).^4^^,^^5^^,^^7^^,^^8^ Previous trials have examined rhGH therapy in CO-GHD adolescents who were treated since childhood using different protocols with controversial benefits.^9^, ^10^, ^11^ Most of these studies that showed bone mineral increase from 14 to 20 years of age occurred at higher weight-dependent doses; thus, little is known about fixed low doses^12^^,^^13^ in younger subjects.

This study aimed to assess the impact of low non-weight-dependent rhGH dose concerning bone accrual in CO-GHD adolescents, who have been treated since childhood. The findings could be important for preventing osteoporotic fractures later in adulthood by optimizing peak bone mass in this vulnerable population.

## Methods

### Participants

This prospective, randomized, open-label, single-center study included adolescents aged 14 to 20 years at the Transition Outpatient Clinic of Instituto da Criança, Hospital das Clínicas, Sao Paulo University Medical School. Participants were recruited between May 2017 and April 2021.

## Inclusion and exclusion criteria

### Inclusion criteria

We selected subjects with GH deficiency in childhood who had been treated with a mean dose of 0.03 mg/kg/day, six times a week, for at least three consecutive years before entering the study^6^. Participants transitioned when their final height was reached, which was defined as growth velocity < 2 cm/year and bone age greater than 14 years in girls and 16 years in boys. All participants were fully pubertal, which was defined as either spontaneous menarche in girls or Tanner IV in boys. If puberty was medically induced, girls were treated with cyclic estrogen/progesterone therapy and boys with 200 mg of testosterone cypionate monthly. Those with hypothyroidism and adrenal insufficiency were treated with levothyroxine and corticosteroids, respectively.

## Exclusion criteria

Patients were excluded if they had chronic diseases, such as chronic renal failure, type 1 *diabetes mellitus*, bone diseases, complex syndromes associated with GH deficiency, and chronic use of corticosteroids, which could alter bone mass.

## Clinical evaluation

After reaching the final height, treatment with rhGH was interrupted for one to three months so that serum levels of somatomedin C (insulin-like growth factor (IGF-I]), C-terminal collagen type I peptide (CTX-I), calcium, phosphorus, alkaline phosphatase (AP), and 25(OH)D could be measured. Bone mineral density (BMD), bone mineral content (BMC), whole-body (WB), and lumbar spine (LS) Z-scores were measured using dual-energy X-ray absorptiometry (DXA). Scores were adjusted for sex, age, and height (Hologic, Discovery W, software 13.5.2.1). BMD and BMC percentage changes were calculated. Low bone mass was considered in cases of LS and WB Z-scores ≤ - 2 SD for sex, age, and height.^14^ Persistence of GH deficiency was defined by baseline IGF-I values lower than ≤ - 2 SD for age and sex, or a GH of less than 5 µg/L based on the peak insulin tolerance test (ITT) as previously described.^13^ An ITT was performed when IGF-I values were between −2 SD and the mean. Those participants who were considered insufficient were allocated into two subgroups using an urn with a paper to each arm. The randomization was simple at a 1:1 ratio: those who discontinued rhGH (GH off) and those who restarted rhGH at a dose of 0.5 mg/day (GH on), six times a week.^13^ The dose was readjusted according to IGF-I concentrations to keep it close to the mean reference values for age and sex.^6^ The insufficient groups were, then, compared to the sufficient subjects (GHS group) After 12 and 18 months of follow-up, all groups’ IGF-I and bone marker values were compared. The percentage changes in LS and WB BMD and BMC were compared at baseline and after 18 months. The enrollment process, follow-up, and allocation of participants to the intervention arm were conducted by VM Kuba throughout the study.

## Patient information

We collected several sets of data: age (years), gender, height (cm), weight (kg), etiology of hormone deficiency, and mean dose of rhGH. Weight was measured using an electronic Filizola scale with a precision of 100 g, and height was measured using a Harpenden Holtain wall stadiometer with a precision of 1 mm.^15^

All patients were asked about their daily calcium intake (mg/day) in each appointment, and instructed to make necessary adjustments so that their daily intake would be 1300 mg/day. If this intake was insufficient, we prescribed calcium carbonate. We also supplemented cholecalciferol if serum concentrations of 25(OH)D were less than 20 ng/mL.^16^ The participants were instructed to do regular physical activity for 150 min weekly. IGF-I, AP, calcium, and phosphorus were measured using a colorimetric method (Cobas C, Roche Hitachi). 25(OH)D was measured by chemoimmunoassay (Cobas E-411, Abbott Park), IGF-I was measured by chemiluminometry (IDS, Immunodiagnostics Systems), and CTX-I was measured using electrochemiluminometry (Elecsys beta-cross Laps/Cobas serum E-411, Roche Diagnostics).

## Ethics approval

The individuals’ identities were protected, and they only participated in the study after the adolescents and their guardians signed informed consent forms. Approval was obtained from the Research Ethics Committee of the University of São Paulo (no. 1511,705), and the study was registered at the WHO Brazilian Clinical Trial Registry REBEC (number UTN U1111- 1280–7723).

## Statistical analysis

With a 5 % significance level and 80 % power, a sample of 42 participants was estimated so that a reduction of one Z-score in the LS or WB BMD could be detected. Assuming a dropout rate of 20 %, we planned to recruit 48 participants. After separating the groups between GH deficient and sufficient, the BMD and BMC percentage changes were used for comparison A Student's *t*-test was used to compare variables with a normal distribution, which were expressed as mean ± stand deviation. A Kruskall–Wallis test was used for those variables without normal distribution, which were expressed as median with a 95 % confidence interval. For comparison between the three groups, a one-way analysis of variance (ANOVA) was used, and between BMD and BMC of GH on and GH off subgroups, a Student's *t*-test was applied. The level of significance was set at *p* < 0.05. Med Calc software version 20.110 was used for data analysis.

## Results

### Patient characteristics

Of the 70 patients recruited, 67 were included in the study, 34 were reclassified as GH sufficient (GHS), and 33 were GH deficient. Of these, 16 were randomized to discontinue rhGH (GH off group), and 17 to restart it (GH on group). Fifty-four patients completed the study (28 patients in GHS, 15 patients in GH off, and 11 patients in GH on). As Supplementary Figure 1 demonstrates, poor adherence was the most common reason for dropping out (five patients in GH and two in GHS). Only one subject in the GH off group dropped out of the study because he moved to another state

The mean participant age was 16.8 ± 1.6 years. Fifty percent were White (27/54), and 51.9 % (28/54) were male. Weights and heights were similar between GH on and GH off (58 ± 10.7 kg and 164.58 cm versus 53.4 ± 15.3 kg and 160.3 ± 11.8 cm, respectively). The average dose of rhGH used by GH was 0.5 ± 0.18 mg/day. In GHS, 100 % had idiopathic and isolated GH deficiency in childhood, in the GH on the group, 54.5 % (6/11) had multiple pituitary hormone deficiency, and 45.5 % had isolated GHD. In the GH off group, 27 % (4/15) had multiple pituitary hormone deficiencies, 53.3 % had isolated GHD, and 20.0 % (2/15) had two hormone deficiencies (Table 1). In the LS BMD group, 18.5 % of participants had a baseline *z* ≤ - 2, and in the WB, 25.9 % (14/54) had a baseline *z* ≤ - 2.

**Table 1:** Demographic characteristics and etiology of the groups in the transition phase.

Data	Group			
	GHS (*N* = 28)	GH On (*N* = 11)	GH Off (*N* = 15)	*p*
Sex				
Male	17 (60.7 %)	9 (81.8 %)	9 (60 %)	0.4479
Female	11 (39.3 %)	2 (18.2 %)	6 (40 %)	
Ethnicity				
African	14 (50 %)	5 (45.5 %)	8 (53.4 %)	1.000
Descent				
Caucasian	14 (50 %)	6 (54.5 %)	7 (46.6 %)	
Weight - median (IQR)	49.50 (42.55–57.17)	58.7 (52.0–65.6)	54.80 (42.45–65.10)	0.297
Height - median (IQR)	156.2 (152.6–162.6)	165.0 (162.2–169.2)	162.0 (156.1–165.2)	0.0886
Initial etiology Isolated ⁎GHD	28 (100 %)	5 (45.5 %):	8 (53.3 %):	
		−1 medulloblastoma	- 1 midline defect	
		- 3 idiopathic	- 3 idiopathic	
		- 1 empty sela	- 2 pituitary hypoplasia	
⁎GHD + another hormone deficiency			- 1 ectopic neuro pituitary	
			- 1 empty sela 3 (20 %)	
			- 2 GHD + hypogonadism (13 %)	
			- 1 GHD + ADH deficiency (6.7 %)	
Multiple pituitary hormone deficiency		6 (54.5 %):	4 (27 %)	
		- 1 pituitary stalk translocation	- 1 absent pituitary stalk	
		2 pituitary	- 1 pituitary hypoplasia	
			- 1 craniopharyngioma	
			1 ectopic neuro-pituitary	
⁎GHD		- hypoplasia - 2 septo - optic dysplasia	- 1 craniopharyngioma	

GHD, GH deficiency.

Statistical analysis: One-way analysis of variance (ANOVA) and Fisher test for categorical variables.

⁎ Used to explain what GHD means.

### Clinical changes

As shown in Table 2, when comparing the three groups after 18 months, a significant difference in the percentage change in the LS BMD between GH off and GHS (0.53 % ± 5.9 and 4.42 % ± 4.1, respectively; *p* < 0.04) was noted. No differences in percentage changes in WB BMD among groups were observed (4.30 %, 1.90 %, and 3.80 % for GH on, GH off, and GHS, respectively; *p* > 0.05).

**Table 2:** Absolute values and percentage variations at baseline and after 18 months of lumbar spine BMD between groups in the transition phase.

LS BMD (g/cm2)	GH on (*n* = 11)	GH off (*n* = 15)	GHS (*n* = 28)
Baseline 95 % CI	0.839 ± 0.11 (0.776–0.914)	0.836 ± 0.11 (0.774–0.899)	0.883 ± 0.14 (0.828–0.939)
18 months 95 % CI	0.867 ± 0.09 (0.803–0.928)	0.848 ± 0.11 (0.785–0.928)	0.922 ± 0.14 (0.866–0.978)
% Variation 95 % CI *p*	3.90 ± 5.0 (0.5 −7.3) > 0.05 vs S	0.53 ± 5.9 (−2.7 – 3.8) > 0.05 vs GH on	4.42 ± 4.1 (2.8 – 5.9) ⁎< 0.04 vs GH off

Statistical analysis: One-way analysis of variance (ANOVA) and Turkey-Kramer multiple comparison tests.

⁎ Used for statistics significance of results.

In the analysis of the GH on and GH off subgroups, the percentage change of LS BMC was significantly higher in GH on (11.02 % ± 10.12 % and 2.05 % ± 10.31 % for GH on and GH off, respectively; *p* < 0.04, F test = 0.736), as shown in Table 3. Although the AP values were similar in the GH off and GH on groups throughout the study, the evolution of CTX-I was different as it decreased over 12 months in GH off and GHS (*p* < 0,05), but not in the GH on group (Table 4). The GHS had IGF-I values higher than both the GH off and GH on groups at baseline (338.8 ± 69.3, 155.0 [95 % CI 44.0–235.0] and 150.82 ± 72.1 ng/dL; *p* < 0.01 for GHS, GH off, and GH on groups, respectively) and at 12 months (319.8 ± 77.5, 179.0 [95 % CI 33.0–290.0, *p* < 0.001) and 193.6 ± 62.09 ng/dL, *p* < 0.01, and at 18 months (309.6 ± 79.1,169.0 [95 % CI 34.0–287.0] and 174.73 ± 52.4 ng/dL; *p* < 0.01). No adverse events were observed.

**Table 3:** Absolute values and percentage variations at baseline and after 18 months of lumbar spine BMC between GH on and GH off subgroups in the transition phase.

LS BMC (g)	GH on (*n* = 11)	GH off (*n* = 15)	*p*
Baseline 95 % CI	45.62 ± 10.21	44.44 ± 13.12	
18 months 95 % CI	49.90 ± 10.54	44.91 ± 12.96	
#% Variation 95 % CI	11.01 ± 10.12	2.02 ± 10.31	⁎< 0.04

# F test for equal variances = 0.736 (95 % IC= −17.865 - −0.117).

⁎ Used for statistics significance of results.

**Table 4:** Evolution of bone markers values at baseline, 12 and 18 months of the groups in the transition phase.

Data	GH off (*n* = 15)	GH on (*n* = 11)	GHS (*n* = 28)
AP (U/L)			
Baseline 95 % IC	107.0 (59.0–288.0)	106.0 (70.0–190.0)	128.6 ± 42.3
12 months	91.0	96.0	104.2 ± 31.5
95 % CI p	(46.0–292.0) > 0.5	(56.0–168.0) > 0.5	> 0.5
18 months	85.0	85.0	93.3 ± 27.6
95 % IC P	(47.0 −390.0) > 0.05	(63.0–160.0) > 0.05	> 0.05
CTX-I (ng/mL)			
Baseline	1.0	1.12 ± 0.31	1.48 ± 0.6
95 % CI	(0.62 - 2.49)		
12 months	0.89	1.04 ± 0.59	1.50 ± 0.5
95 % CI *p*	(0.48 −2.37) ⁎< 0.05	> 0.05	⁎< 0.05
18 months	0.81	1.03 ± 0.42	1.13 ± 0.4
95 % CI *p*	(0.24- 2.19) > 0.05	> 0.05	> 0.05

Statistical analysis: Nonparametric repeated measures (ANOVA).

⁎ Used for statistics significance of results.

## Discussion

Our results indicate that somatropin withdrawal for 18 months limited the bone gain in the lumbar spine of CO-GHD adolescents in transition who had undergone treatment since childhood. The BMC increased by 11.01 % in those who were on somatropin at a non-weight-based dose versus a non-significant increase of 2.02 % in those who discontinued it. Furthermore, the GH off group also had much lower BMD than that of the GHS (0.5 % and 4.42 %, respectively). Most studies that showed bone mineral accrual used higher weight-based doses.^17^, ^18^, ^19^, ^20^ Some were retrospective,^21^^,^^22^ and not all of them had a control group.^22^^,^^23^ As far as we know, our study is the first study to perform non-weight-based low-dose somatropin in a younger cohort with a mean age of 16.8 years as most research with this treatment regimen has been in populations over 18 years of age.^20^^,^^24^^,^^25^

The impact of several interventions on bone mass is quite variable due to the heterogeneity of study designs, rhGH doses used in childhood and the transition period, duration of treatment breaks, and differences in body development that normally occur in adolescence.^17^^,^^24^^,^^26^

The somatropin doses used during childhood and the transition period may have influenced our results. Mauras et al. showed no gain in CO-DGH patients with a mean age of 15.8 years after two years of treatment at 20 µg/kg/day during the transition. However, this population had already been using 40 µg/kg/day since childhood. Such treatment could have optimized bone accrual during growth before reaching the final height, leaving the Z-score equal to or greater than the average for sex and age.^27^ Diverging from this study, our cohort was treated with an average dose of 30 µg/kg/day in childhood; 18.5 % reached the transition presenting baseline spine BMD Z-score of ≤ −2, and 25.9 % (14/54) in the WB. In another survey, although the rhGH dose was the same in childhood as ours, no bone loss was reported after two years of withdrawal during the transition period. However, these patients were treated longer since the average age of entry into the study was 19 years, thus explaining the greater acquisition of bone mass.^23^

Regarding the duration of treatment withdrawal, the results are conflicting. Fors et al. found no loss two years after discontinuation,^23^ while Drake et al. still detected it after one year.^18^ Nevertheless, it is noteworthy that the population of the first study was older than that of the last one when the treatment break occurred (mean age of 19 versus 17 years old). These different results can both be explained by a persistent action of rhGH on bone mass after withdrawal when the final height is reached. It is believed that somatropin triggers a cycle of long-lasting bone remodeling, even if the patient is no longer exposed to it, but to be effective, treatment reinstitution has to be done between 14 and 17 years of age, which was done in our cohort. Therefore, it seems that not only the duration of the pause but also the age at which somatropin is restarted influences bone accrual.

Although the reference values for the markers of bone turnover (AP and CTX) are not well established in the pediatric group, they may indicate an increase in early bone formation or resorption.^28^^,^^29^ Despite similar AP values in both GHD groups, a decrease in CTX-I at 12 months in those who discontinued treatment occurred, suggesting a decrease in bone remodeling. On the other hand, in the group that restarted it, both markers were stable until 18 months, which indicates that even at a low dose, somatropin was efficient in maintaining bone mineral accrual. Concerning GHS, a reduction in both markers at 12 months occurred, suggesting an adaptive response of the bone to the abrupt withdrawal of the treatment, which remained stable, thus ensuring bone accrual. This finding is in line with those describing DXA results after 18 months of follow-up which showed a gain in spine BMC in those who maintained rhGH in comparison to those who did not (11.01 % and 2.2 %, respectively) in addition to spine BMD in GHS (4.42 % versus 0.5 in the GH off group). These results are similar to those of Baroncelli et al., who observed that the peak spine BMD occurred one to three years after the final height was reached and was delayed in the CO- GHD compared to that in GH-sufficient subjects. This finding showed that GH/IGF-I plays an important role in both the acquisition and maintenance of bone mass in the spine in GHD.^30^

In this study, the adequate daily intake of calcium and vitamin D was ensured in all participants, so no interference of these factors with the expected outcomes occurred.

Another strength relates to the study's prospective design, which included GH on and off-insufficient groups. Lee et al. followed adolescents who restarted rhGH at around 18 years of age. As there was a Z-score increase only in the femur BMD, the lack of response in LS and a GHD off group made it difficult for them to attribute this improvement to treatment reinstitution.^22^ The analysis of BMD Z-score in conjunction with percentage change also made the DXA more sensitive for diagnosing the bone accrual in our research. Analysis of the Z-score alone may have limited the conclusions of others, who did not find any increase in bone accrual after rhGH reinstitution.^22^^,^^27^

This study has some limitations. The study was a one-center study in which 54 individuals mainly from the State of Sao Paulo were followed for 18 months, which requires caution when extrapolating the results to other regions of the country. We would like to comment that despite the large dispersion of the percentage change values in LS BMC in this sample of GHD groups, statistical tests showed that a normal distribution was found, and this variability was similar in the two groups as demonstrated by F test for variances (*p* = 0.735). This finding explains the statistical significance of the difference between the means, even with a high standard deviation value. Finally, we could not follow the evolution of bone mass and osteo-metabolic profiles since the beginning of treatment in childhood as the cohort arrived at the transition outpatient clinic after reaching their final heights.

Most guidelines recommend assessing bone mass using DXA during the transition period, and then, only two to five years after suspending or restarting rhGH.^12^^,^^13^ They also advise discontinuing the treatment in those who present isolated GHD or in those with an additional pituitary hormone deficiency. However, as many CO-DGH patients reaching the transition have a low bone mass, we suggest that DXA and bone markers be performed during rhGH treatment at the beginning of puberty and during the transition. If the spine z score is <−2 after reaching the final height, the treatment should be maintained until the end of peak bone mass regardless of GHD etiology, and then monitored using semi-annual measurements of bone markers and an annual DXA.

In conclusion, somatropin withdrawal limits one from reaching peak spine BMD in CO-GHD in transition, suggesting that the treatment should continue, especially in those with low bone mass.

## Conflicts of interest

The authors declare no conflicts of interest.

## Appendix Supplementary materials

Image, application 1
