# Supplementary material for: Impact of non-weight-dependent low-dose somatropin on bone accrual in childhood-onset GH deficient in the transition: an 18-month randomized controlled trial
Source: J Pediatr (Rio J). 2025 Jan 2;101(2):255–61. doi: 10.1016/j.jped.2024.10.010 (PMC11889693; doi:10.1016/j.jped.2024.10.010)

**JPED-D-24-00247 – Supplementary Material**

**Figure 1 Supplementary** Flowchart of the participants of the study.


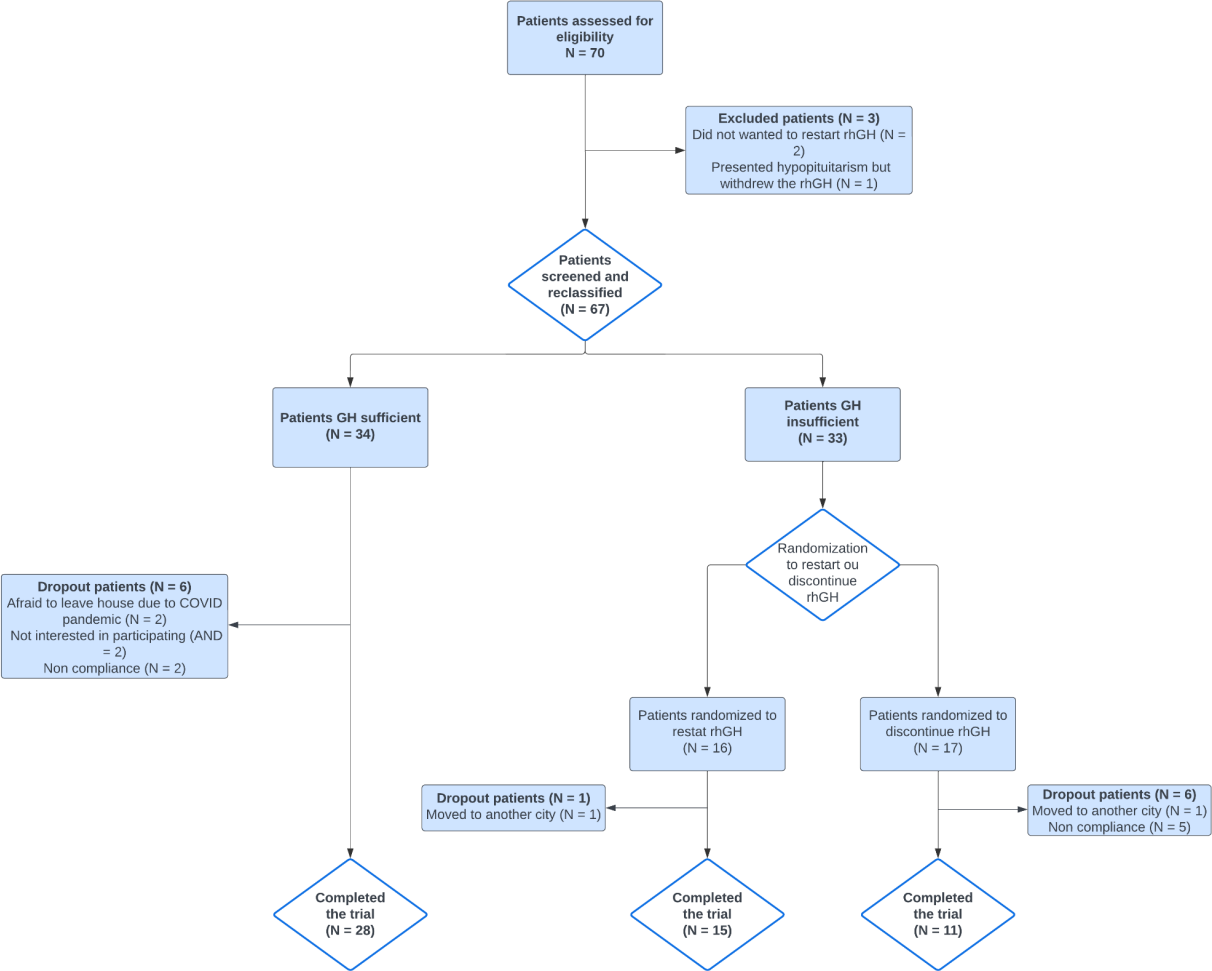

Supplement: Supplementary file 1 [file mmc1.docx]
